# Risk factors and severity of functional impairment in long COVID: a single-center experience in Croatia

**DOI:** 10.3325/cmj.2022.27

**Published:** 2022-02

**Authors:** Marko Banić, Mateja Janković Makek, Miroslav Samaržija, Davorka Muršić, Zagorka Boras, Vesna Trkeš, Denis Baričević, Marta Koršić, Latinka Basara, Tajana Jalušić, Glunčić, Andrea Vukić Dugac

**Affiliations:** 1 Clinic for Respiratory Diseases “Jordanovac,” University Hospital Center Zagreb, Zagreb, Croatia; 2 University of Zagreb School of Medicine, Zagreb, Croatia

## Abstract

**Aim:**

To determine the frequency of common symptoms in long COVID and their effect on the quality of life, and to determine the factors contributing to a more severe long COVID.

**Methods:**

The study enrolled 266 patients who were either referred to long-COVID outpatient clinic or were inpatients undergoing rehabilitation. The data were collected between December 2020 and May 2021. We evaluated the symptoms experienced during acute and long COVID and comorbidities. Functional status was assessed with Post Covid Functional Status (PCFS).

**Results:**

The final sample consisted of 261 patients. After acute COVID-19 period (>4 weeks), almost 80% of patients had impaired functional status. Only 21.5% reported no functional impairment (0 on PCFS scale). A higher PCFS score was associated with female sex (*P* < 0.001) and oxygen therapy requirement during acute disease (*P* = 0.001). However, it was not associated with having a pre-existing lung disease (*P* = 0.749). Disease severity did not pose a risk for developing a more severe long COVID.

**Conclusion:**

Women were at greater risk for developing greater functional impairment in long COVID, although we have no explanation why. Malignant disease and hypertension also presented a risk factor for greater functional impairment. More studies are warranted to determine if patients with certain lung disease are more susceptible to long COVID.

Coronavirus disease-2019 (COVID-19), resulting from a novel severe acute respiratory syndrome coronavirus 2 (SARS-CoV-2), has imposed an immense burden on health care systems (1,2). Complex pathophysiologic mechanisms of COVID-19 in the most severely ill patients include lung injury, endotheliopathy, vasculopathy, and severe immunologic dysregulation (3-6). The most severe forms of COVID-19 are acute respiratory distress syndrome and cytokine storm (7).

A number of patients recovering from acute COVID-19 have persistent or newly developed symptoms affecting their quality of life (8). Beside physical symptoms such as fatigue, cough, chest pain, and headaches, there are persisting psychological problems including insomnia, short-term memory loss, anxiety, and depression (9-12). Long COVID may manifest as respiratory, neurological, cardiovascular, or psychological symptoms, but new manifestations are increasingly being described, such as post-COVID cholangiopathy (13-17). Proposed pathophysiological pathways mainly include tissue damage by hypoxia and long-lasting inflammatory process, but further research on these topics is warranted (15). Studies have confirmed that long COVID affects individuals regardless of acute COVID-19 severity and may involve multiple organs (15). There is a lack of nomenclature for syndromes occurring after acute COVID-19 (18).

The National Institute for Health and Care Excellence (NICE) guidelines for managing the long-term effects of COVID-19 used in this study define two periods after the acute illness. Ongoing symptomatic COVID-19 lasts from 4 to 12 weeks after the initial symptoms, and post-COVID-19 syndrome lasts for more than 12 weeks after the initial symptoms. Both of these periods fall under the term “long COVID“ (19). Long COVID symptoms include loss of smell and taste, fatigue, muscle weakness, shortness of breath, hair loss, sleep difficulties, diarrhea, and others (8,19). Several guidelines have been published about long COVID treatment, mainly therapy guidelines for complications of acute COVID-19, such as thrombotic events and lung fibrosis (19,20). Clinical trials examining the effectiveness of hyperbaric oxygen therapy, montelukast, and physical therapy in long COVID are under way (20).

In an attempt to stratify patients with long COVID into severity groups, Klok et al (21) proposed a simple 0-4 Post-COVID Functionality Status (PCFS) scale for quick assessment of patients’ functional status over the previous week. Simple to use and widely applicable, PCFS scale covers a wide range of functional limitations, including lifestyle changes and social activity limitations. A flowchart facilitates the status assessment (Figure 1) (21). PCFS score can also be used for determining the quality of life after acute COVID-19 (22), showing better properties than other quality-of-life questionnaires and scales. This scale could be useful in long-term evaluation and follow-up of long COVID patients.

**Figure 1:**
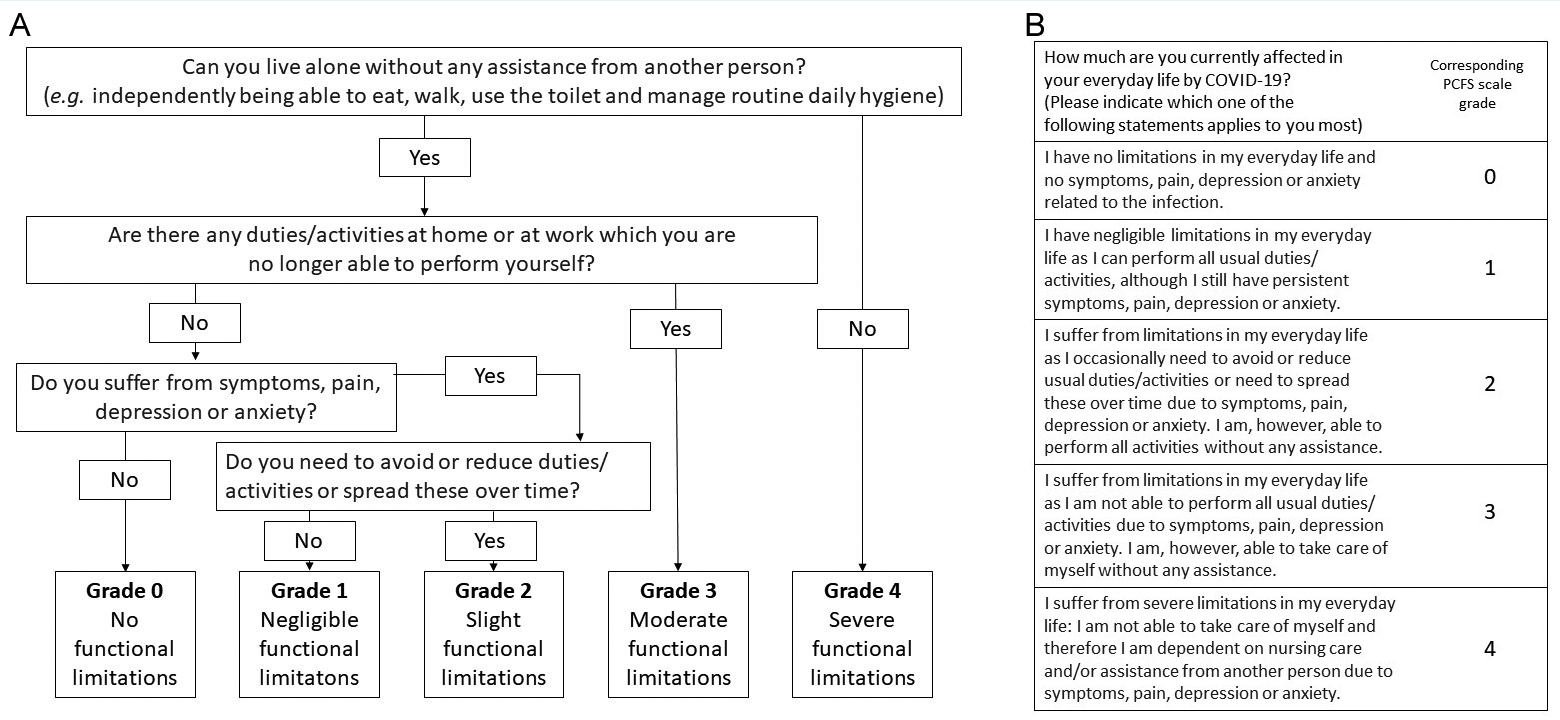
A flowchart (**A**) and description chart (**B**) used in determining patient’s Post-COVID-19 Functional Status (PCFS) score, developed by Klok et al (21). Adapted and used with the author’s permission.

Different chronic lung conditions lead to different outcomes in acute COVID-19. Patients with interstitial lung diseases (ILD) had a poor outcome, greater ICU admittance, and poorer outcomes than patients without ILD (23). Patients with obstructive lung disease showed mixed results. Chronic obstructive lung disease (COPD) led to poorer COVID-19 outcomes, but the same was not observed for asthma (24-26). An ambidirectional cohort study from Wuhan showed that 76% of all patients diagnosed with COVID-19 had some symptoms of long COVID 6 months after discharge. However, a relatively small number of these patients had a pre-existent pulmonary condition (8). A few studies so far have analyzed the epidemiology and symptoms of long COVID. To our knowledge, no long-COVID studies in Croatia used PCFS to assess the functional status of patients. We aimed to investigate whether age, sex, comorbidities, and severity of acute COVID-19 present a risk for developing more severe long COVID.

## PATIENTS AND METHODS

### Patients

This cross-sectional study involved patients examined in the newly formed long-COVID outpatient clinic and inpatient ward at the Clinic for Respiratory Diseases Jordanovac, University Hospital Center Zagreb. We enrolled patients whose symptoms persisted after acute COVID-19 illness and who experienced some new symptoms attributable to long COVID. The patients were examined 4 weeks up to 8 months after the onset of acute COVID-19. The patients were referred to our clinic by their general practitioners or were referred for follow-up after discharge from any hospital in Zagreb where they were treated for COVID-19. All the patients had previous SARS-CoV-2 infection confirmed by laboratory polymerase chain reaction testing. The exams were performed from December 2020 to May 2021. The study was approved by the Ethics Committee of University Hospital Center Zagreb (02/21 AG). Patients signed a written informed consent for study participation.

### Methods

We measured patients' vital parameters and noted their comorbidities. We collected data on symptoms experienced during and after the acute disease (>4 weeks after the onset). The intensity of each symptom was not graded, but we used PCFS scale scoring from 0-4 (21).

The interviewer used the questions from the PCFS flowchart (Figure 1) to determine the category to which the patient belongs. The description chart was used as an aid. Long COVID symptoms examined were breathlessness or shortness of breath, dry cough, fatigue, headache, muscle weakness, fever, rash, and psychological symptoms. We established whether the patients were treated as outpatients, ward inpatients, or intensive care unit inpatients. We also collected data on the need for oxygen therapy (conventional oxygen therapy via nasal cannula/mask or mechanical ventilation). Data about corticosteroid therapy both in the acute and long-COVID period were also obtained. All the information was collected either through interviews with patients or by examining their medical documentation.

### Statistical analysis

The normality of distribution was assessed with the Shapiro-Wilk test. Categorical data are presented as counts and percentages, and continuous data are presented as means and standard deviations. The Kruskal-Wallis test with the Dunn post-hoc test was used to analyze PCFS values across different levels of treatment. The Mann-Whitney-Wilcox U test was used in other two-group comparisons for ordinal-value PCFS. A p value (two-sided)<0.05 was considered significant. We had no missing data. The analysis was performed with R studio (27).

## RESULTS

Of all patients examined in our long-COVID outpatient clinic, 266 agreed to participate. Five patients were excluded (their follow-up was either <4 weeks or >8 months), which left 261 patients in the final sample. The median age was 53 years (range, 20-86 years). There were 123 female patients (47.1%). Most of the patients were referred to the long-COVID clinic by their general practitioner solely because of persisting symptoms (203 patients, 77.8%). A smaller portion of the patients came to their regular follow-ups because of chronic pulmonary conditions (COPD, asthma, ILD, and others) but they also had long COVID symptoms (43 patients, 16.5%). Fewest patients (15 of them, 5.7%) were admitted to hospital after a long and difficult course of acute COVID-19 because of prolonged oxygen supplementation dependency or the need for pulmonary rehabilitation.

During the acute COVID-19 phase, 185 (70.9%) patients were treated as outpatients, 63 were treated on COVID-19 wards (24.1%), and 13 were treated in the ICU at some point of their hospital stay (5.0%) (Table 1).

**Table 1:** The associations of Post-COVID-19 Functional Status (PCFS) scores and patients' characteristics. Data are count (%) unless otherwise specified

			PCFS^‡^					
	Total sample^†^	0		1	2	3	4	P
General data*								
Total sample	261 (100)	56 (21.5)		76 (29.1)	72 (27.6)	52 (19.9)	5 (1.9)	
Female	123 (47.13)	11 (8.9)		37 (30.1)	39 (31.7)	34 (27.6)	2 (1.6)	<0.001
Outpatients	185 (70.9)	41 (22.2)		60 (32.4)	52 (28.1)	32 (17.3)	0 (0.0)	
Ward patients	63 (24.1)	13 (20.6)		13 (20.6)	18 (28.6)	16 (25.4)	3 (4.8)	
Intensive care unit patients	13 (5.0)	2 (15.4)		3 (23.1)	2 (15.4)	4 (30.8)	2 (15.4)	
Oxygen therapy	61 (23.4)	10 (16.4)		11 (18.0)	17 (27.9)	18 (29.5)	5 (8.2)	0.001
Mechanical ventilation	2 (0.8)	0 (0)		0 (0)	0 (0)	1 (50)	1 (50)	0.027
Days since the onset of COVID-19 to follow-up^§^	88.7 ± 43.6	77.8 ± 35.3		94.6 ± 45.5	92.2 ± 46.8	85.9 ± 42.3	101.6 ± 55.4	
Age^§^	53.6 ± 14.2	55.5 ± 14.6		50.6 ± 14.3	51.9 ± 12.6	57.1 ± 14.4	65.2 ± 15.0	0.01
Number of symptoms^§^	2.9 ± 2.0	0.5 ± 0.9		2.6 ± 1.5	3.8 ± 1.5	4.4 ± 1.8	5.2 ± 1.1	<0.001
**Comorbidities**								
None	123 (47.1)	28 (50.0)		40 (52.6)	32 (44.4)	22 (42.3)	1 (20.0)	
Hypertension	106 (40.6)	21 (37.5)		25 (32.9)	34 (47.2)	23 (21.7)	3 (60.0)	0.012
Diabetes type 2	35 (13.4)	7 (12.5)		7 (9.2)	10 (13.9)	9 (17.3)	2 (40.0)	0.933
Active malignant disease	17 (6.5)	2 (3.6)		7 (9.2)	3 (4.2)	3 (5.8)	2 (40.0)	0.041
Coronary artery disease	14 (5.4)	2 (3.6)		3 (3.9)	4 (5.6)	5 (9.6)	0 (0.0)	0.433
Atrial fibrillation	9 (3.4)	2 (3.6)		3 (3.9)	0 (0.0)	4 (7.7)	0 (0.0)	0.623
Solid organ transplant	3 (1.1)	1 (1.8)		0 (0.0)	1 (1.4)	1 (1.9)	0 (0.0)	0.164
Chronic kidney disease	1 (0.4)	0 (0.0)		0 (0.0)	1 (1.4)	0 (0.0)	0 (0.0)	0.612
Rheumatoid arthritis	7 (2.7)	1 (1.8)		1 (1.3)	2 (2.8)	3 (5.8)	0 (0.0)	0.436
Inflammatory bowel disease	1 (0.4)	0 (0.0)		0 (0.0)	0 (0.0)	1 (1.9)	0 (0.0)	0.612
**Respiratory comorbidities**								
Chronic obstructive pulmonary disease	9 (3.4)	4 (7.1)		0 (0.0)	1 (1.4)	3 (5.8)	1 (20.0)	0.853
Asthma	33 (12.6)	5 (8.9)		12 (15.8)	9 (12.5)	6 (11.5)	1 (20.0)	0.486
Lung cancer	1 (0.4)	0 (0.0)		1 (1.3)	0 (0.0)	0 (0.0)	0 (0.0)	0.168
Bronchiectasis	1 (0.4)	0 (0.0)		0 (0.0)	1 (1.4)	0 (0.0)	0 (0.0)	0.612
Sarcoidosis	4 (1.5)	1 (1.8)		1 (1.3)	1 (1.4)	1 (1.9)	0 (0.0)	0.343
Interstitial lung disease	4 (1.5)	0 (0.0)		0 (0.0)	1 (1.4)	2 (3.8)	1 (20.0)	0.622
Cystic fibrosis	1 (0.4)	1 (1.8)		0 (0.0)	0 (0.0)	0 (0.0)	0 (0.0)	0.169
Active lung tuberculosis	1 (0.4)	1 (1.8)		0 (0.0)	0 (0.0)	0 (0.0)	0 (0.0)	0.174
**Acute COVID-19 symptoms**								
Fever	173 (66.3)	34 (60.7)		51 (67.1)	49 (68.1)	34 (65.4)	5 (100.0)	0.357
Anosmia or ageusia	141 (54.0)	21 (37.5)		46 (60.5)	37 (51.4)	33 (63.5)	4 (80.0)	0.021
Dry cough	178 (68.2)	29 (51.8)		54 (71.0)	49 (68.1)	42 (80.8)	4 (80.0)	0.006
Breathlessness	173 (66.3)	17 (30.4)		43 (56.6)	60 (83.3)	49 (94.2)	4 (80.0)	<0.001
Chills	126 (48.3)	22 (39.3)		33 (43.4)	36 (50.0)	30 (57.7)	5 (100.0)	0.01
Headache	133 (51.0)	13 (23.2)		41 (53.9)	42 (58.3)	36 (69.2)	1 (20.0)	<0.001
Myalgia	165 (63.2)	23 (41.5)		53 (69.7)	49 (68.1)	37 (71.1)	3 (60.0)	0.005
Diarrhea	70 (26.8)	10 (17.9)		19 (25.0)	21 (29.2)	17 (32.7)	3 (60.0)	0.027
**Long COVID symptoms**								
Dry cough	82 (31.4)	9 (16.5)		30 (39.5)	22 (30.6)	19 (36.5)	2 (40.0)	0.082
Breathlessness	152 (58.2)	3 (5.4)		43 (56.6)	59 (81.9)	43 (82.7)	4 (80.0)	<0.001
Fatigue	177 (67.8)	9 (16.1)		48 (63.2)	66 (91.7)	50 (96.1)	4 (80.0)	<0.001
Chest pain	62 (23.7)	3 (5.4)		16 (21.0)	22 (30.6)	19 (36.5)	2 (40.0)	<0.001
Muscle weakness	84 (32.2)	3 (5.4)		18 (23.7)	30 (41.7)	30 (57.7)	3 (60.0)	<0.001
Fever	24 (9.2)	1 (1.8)		2 (2.6)	7 (9.7)	13 (25.0)	1 (20.0)	<0.001
Rash	17 (6.5)	1 (1.8)		4 (5.3)	5 (6.6)	6 (11.5)	1 (20.0)	0.023
Psychological symptoms	32 (12.3)	0 (0.0)		9 (11.8)	12 (16.7)	10 (19.2)	1 (20.0)	0.047

*All percentages in this section (except for “Total sample” column) represent a fraction of the respective row’s total sample population.

†All percentages in the “Total sample” column represent a fraction of whole study population.

‡Each percentage in PCFS columns represents a fraction of the respective score’s total population, except in the “General data” section.

§Mean ± standard deviation.

Of 76 patients who were treated as inpatients, 61 received oxygen supplementation therapy – either conventional or high-flow oxygen and only 2 were intubated and mechanically ventilated.

**Figure 2:**
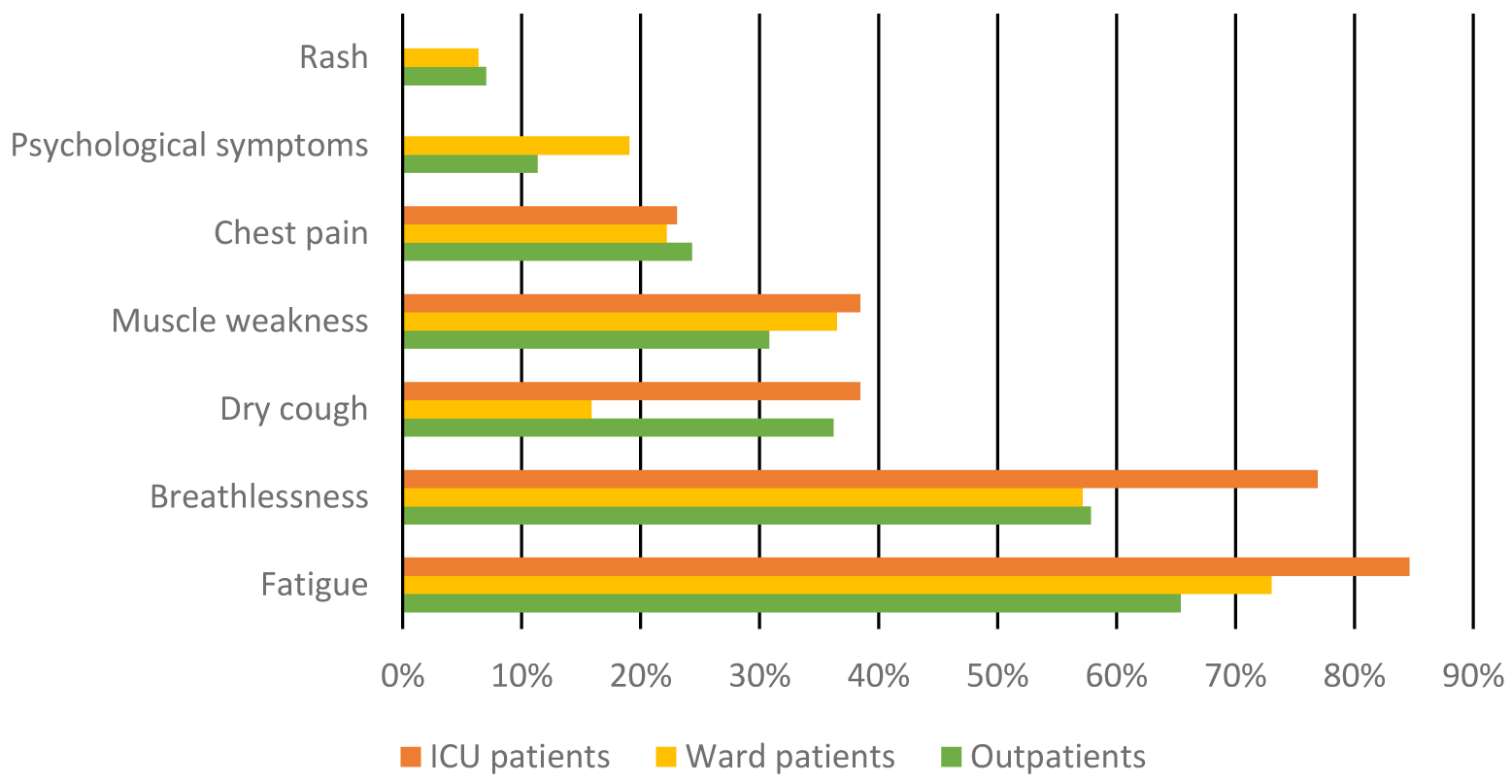
The percentage of patients undergoing different treatment levels (outpatients, ward patients, intensive care unit [ICU] patients) who reported specific Post-COVID-19 Functional Status (PCFS) score. Horizontal axis: PCFS score.

The exams were performed between 28 and 222 days after the onset of acute COVID-19 (mean 88.7 ± 43.6). The most frequent symptoms during acute COVID-19 were cough, fever, dyspnea, and myalgia, reported by more than 60% patients. Nearly half of the patients reported anosmia, headache, and chills (Figure 2). At the exam, 38 patients (14.6%) experienced no symptoms related to long COVID and 137 patients (52.5%) reported three or more symptoms. A total of 112 patients (42.9%) were treated with corticosteroids at some point during acute COVID-19 or in the long-COVID period.

The most common symptoms of long COVID included fatigue (68.2%) and dyspnea (58.6%). The least common symptoms were psychological symptoms (12.6%), fever (9.2%), and rash (6.5%) (Table 1).

**Figure 3:**
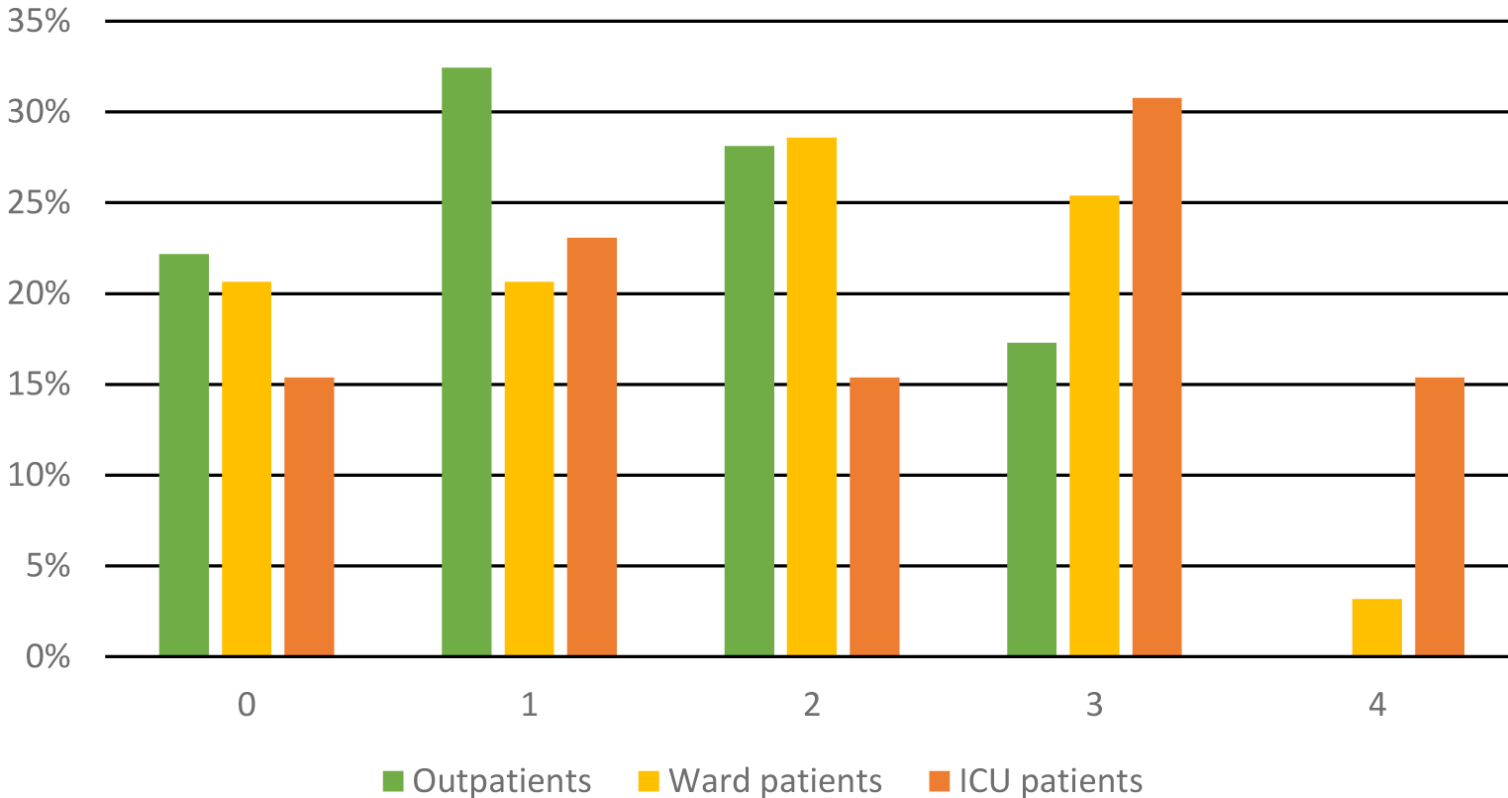
The percentage of patients experiencing long-COVID symptoms in groups that underwent different levels of treatment.

PCFS score 0 was reported by 56 patients (21.5%). Seventy-six reported score 1 (29.1%). Only five patients reported score 4 (1.9%). Outpatients most often reported score 1 (32.4% of all outpatients), while ward-treated patients most often reported score 2 (28.6% of all ward-treated patients). The ICU-treated patients most often reported score 3 (30.4%). No outpatients and fewest ward-treated patients reported score 4. ICU-treated patients were least likely to report scores 0 and 2 (Figure 3). The three patient groups significantly differed in the mean PCFS score (*P* = 0.045). However, *post-hoc* analysis did not confirm a significant difference between the groups (Dunn test, ICU-outpatient Z = 1.845, *P* = 0.065; ICU-ward Z = 0.830, *P* = 0.406; and outpatient-ward Z = -1.895, *P* = 0.058).

PCFS score was associated with the number of symptoms experienced during long COVID (*P* value <0.001). Patients requiring any form of oxygen therapy reported overall higher PCFS scores than those who did not require oxygen therapy (*P* < 0.001). Women experienced more symptoms and reported higher PCFS score than men (*P* < 0.001). Age was also a significant factor, resulting in higher PCFS scores (*P* = 0.01).

Most common comorbidities were hypertension (106 patients, 40.6%), type-2 diabetes (35 patients, 13.4%), coronary arteries disease (14 patients, 5.4%), and atrial fibrillation of any kind (9 patients, 3.4%). Less common comorbidities included chronic kidney disease, rheumatic and immunologic diseases, inflammatory bowel disease, psychiatric illnesses, and immunosuppression due to solid organ transplant. Seventeen patients had a malignant disease, either in active treatment or in their medical history. Arterial hypertension and active malignant disease were the only comorbidities associated with a higher PCFS score (*P* = 0.012 and *P* = 0.043, respectively). However, this may be due to a small sample size and a significant portion of younger patients.

Fifty-four patients (20.7%) had a chronic lung condition – either COPD, asthma, interstitial lung disease, cystic fibrosis, pulmonary sarcoidosis, lung carcinoma in treatment, pulmonary tuberculosis, or other. A total of 18.4%, 18.1%, 23.1%, and 60% of the patients reporting scores 1, 2, 3, and 4, respectively, also had a chronic lung condition. Although the proportion of patients who had a chronic lung condition was higher among patients with a high PCFS score (3 or 4), PCFS score was not associated with the presence of a preexisting lung condition (*P* = 0.723) (Table 1).

## DISCUSSION

Our study showed that female sex and oxygen therapy predisposed to a more severe long COVID. Although the patients with different treatment levels differed in PCFS scores, the difference did not reach the level of significance. The number of patients who reported the highest score was low and it included many ICU-treated patients, who experienced prolonged hospital stay and heavy or invasive medical treatment and had long-term health issues regardless of COVID-19 (28). Our data were collected during a period when literature on long COVID was scarce. In our study, patients requiring oxygen therapy also reported higher PCFS scores. Lung injury severe enough to cause hypoxemia may prolong the recovery and be an important factor in long COVID. Interestingly, women reported higher PCFS scores than men. Sudre et al (29) also reported female sex to be a risk factor for developing long COVID, but could not establish with certainty whether it was also a risk factor for developing more symptoms and more severe long COVID with greater functional impairment. A retrospective cohort study on over 273 000 patients (including COVID-19 cohort and influenza cohort) (30) reported a higher incidence of long COVID symptoms in women and young adults (unlike the previously mentioned study where older age was a risk factor for prolonged COVID-19 symptoms) but also did not assess the effects of these symptoms on the quality of life. A report from another tertiary center in Croatia showed younger men (<57 years of age) having better survival outcomes in acute COVID-19 than women of the same age group. In addition, men older than 57 years had worse survival outcomes than women in the same age group (31). It is not known whether this applies to the long-COVID period as well. In our study, men and women did not significantly differ in age, while both younger and older patient groups reported higher PCFS scores.

In a study by Carfi et al (11), fewer patients than in our study reported fatigue (53.1% vs 68.2%), dyspnea (43.4% vs 58.6%), and cough as persisting symptoms (less than 20% vs 31.4%). We and Carfi et al found similar number of participants with no symptoms – 12.6% compared to 14.6%, respectively. In the study by Carfi et al, all the patients were hospitalized for acute COVID-19 treatment, and the follow-up after the onset of COVID-19 was shorter (60.3 days). However, Carfi et al did not examine psychological symptoms such as insomnia, short-term memory loss, and “brain fog.“ The observed differences between the studies may be explained by the difference in studied population – 9.1% of the patients in the study by Carfi et al had COPD (the only pulmonary comorbidity examined), as opposed to 20.7% of our patients. This could account for the greater percentage of patients reporting dyspnea and cough as persisting symptoms. Carfi et al also included only hospitalized patients.

Halpin et al (32) showed comparable results to our findings regarding the number of patients experiencing symptoms during the long COVID period. However, they examined ward-treated and ICU-treated patients. As opposed to Halpin et al, we did not differentiate between different psychological issues, such as memory loss, insomnia, or lack of concentration. The rates of psychological symptoms reported in our study may have been higher if each of those were specified. However, ward-treated patients in the study by Halpin et al less frequently reported dry cough.

In our study, PCFS scores did not significantly differ between the groups of patients with COPD, asthma, or without any previously known lung condition. The number of patients with each lung disease in this study was not large, and further studies are needed to determine which of the lung disease pose risk for developing more severe long COVID. Furthermore, the COVID-19 pandemic reduced the overall individual mobility (33), but it is unknown whether patients with chronic conditions adhered more to the “stay-at-home” recommendation. There is an ongoing study on the habits of COPD patients in our Center, which may investigate this more issue more closely.

PCFS is a subjective score that depends greatly on the patient’s subjective impression of the long COVID manifestation. Although a flow-chart may be helpful in determining to which group the patient belongs, patients with previous lung or heart disease may not report higher PCFS scores. The score may not provide valuable data when describing discomfort in patients with chronic lung disease since some symptoms, such as cough and dyspnea, already match the symptoms of the chronic disease.

Limitations of this study include a single-center experience, a possible selection bias, small number of participants, as well as a small number of patients in the chronic lung conditions group and ICU-treated patient group. Recall bias may have also occurred, especially for patients with later follow-up dates. Response bias may have been minimized, since the flow-chart helps the patients choose the right category. Furthermore, we did not differentiate between psychological problems in long COVID, which may account for underreporting of these symptoms. To clarify the effect of COVID-19 and long COVID severity in different patient groups, studies with more participants need to be conducted, especially those involving patients with different lung diseases. We did not examine a sample of all the patients who had COVID-19, but only of patients referred to our institution who experienced one or more symptoms that could be attributed to a recent COVID-19 infection. This may have led to selection bias. Based on these data, it cannot be determined whether the proportion of patients experiencing long COVID but not requiring hospital admission was greater than the proportion of patients experiencing long COVID and requiring admission.

In conclusion, women reported significantly greater quality-of-life impairments after acute COVID-19. Arterial hypertension and active malignant disease were independently associated with higher functional impairment in long COVID. We cannot explain why even people with mild disease may have prolonged disabilities or why female sex presents a risk factor for developing worse functional impairment. In addition, patients suffering from pre-existing lung conditions did not display more intense long COVID symptoms than patients without these conditions. Further research including more patients from each subgroup of chronic lung conditions is warranted to determine which lung pathology is associated with long COVID effects. This study helped us determine which patients were more likely to suffer from long COVID and could be of aid to physicians dealing with these patients.
